# Clinical implementation of Dosimetry Check™ for TomoTherapy^®^ delivery quality assurance

**DOI:** 10.1002/acm2.12480

**Published:** 2018-10-24

**Authors:** Eunah Chung, Dongyeol Kwon, Taeyang Park, Hyeri Kang, Yoonsun Chung

**Affiliations:** ^1^ Department of Radiation Oncology Samsung Medical Center Sungkyunkwan University School of Medicine Seoul Korea; ^2^ Department of Nuclear Engineering Hanyang University Seoul Korea

**Keywords:** delivery quality assurance (DQA), Dosimetry Check™, pretreatment verification, TomoTherapy^®^

## Abstract

**Purpose:**

The delivery quality assurance (DQA) of intensity‐modulated radiotherapy (IMRT) plans is a prerequisite for ensuring patient treatments. This work investigated the clinical usefulness of a new DQA system, Dosimetry Check™(DC), on TomoTherapy^®^‐based helical IMRT plans.

**Methods:**

The DQA was performed for 15 different TomoTherapy^®^‐based clinical treatment plans. In Tomotherapy^®^ machines, the couch position was set to a height of 400 mm and the treatment plans were delivered using QA‐Treatment mode. For each treatment plan, the plan data and measured beam fluence were transferred to a DC‐installed computer. Then, DC reconstructed the three‐dimensional (3D) dose distribution to the CT images of the patient. The reconstructed dose distribution was compared with that of the original plan in terms of absolute dose, two‐dimensional (2D) planes and 3D volume. The DQA results were compared with those performed by a conventional method using the cheese phantom with ion chamber and radiochromic film.

**Results:**

For 14 out of the 15 treatment plans, the absolute dose difference between the measurement and calculation was less than 3% and the gamma pass rate with the 3%/3 mm gamma evaluation criteria was greater than 95% for both DQA methods. The *P*‐value calculated using Wilcoxon signed‐rank test was 0.256, which implies no statistically significance in determining the absolute dose difference between the two methods. For one treatment plan generated using the 5.0 cm field width, the absolute dose difference was greater than 3% and the gamma pass rate was less than 95% with DC, while the DQA result with the cheese phantom method passed our TomoTherapy^®^ DQA tolerance.

**Conclusion:**

We have clinically implemented DC for the DQA of TomoTherapy^®^‐based helical IMRT treatment plans. DC carried out the accurate DQA results as performed with the conventional cheese phantom method. This new DQA system provided more information in verifying the dose delivery to patients, while simplifying the DQA process.

## 1 INTRODUCTION

Modern radiotherapy has become more complicated in its quest to deliver a highly conformal dose to a defined target volume, while sparing organs at risk near the target volume. The TomoTherapy^®^ (Accuray, Sunnyvale, CA, USA) is one of the modern radiotherapy systems allowing a continuous dose delivery in a helical fashion around the anatomical site to be treated. The quality assurance (QA) of dose delivery using TomoTherapy^®^ is a prerequisite for ensuring patient treatments.

For TomoTherapy^®^‐based intensity‐modulated radiotherapy (IMRT), the current delivery quality assurance (DQA) process consists of comparing measured versus calculated doses in a phantom using an ionization chamber and a film^1^, ^2^ or using detector array devices.^3^, ^4^, ^5^, ^6^ These devices have generally provided such accurate DQA results in terms of low absolute dose difference or high gamma pass rate. However, these DQA processes are also time‐consuming and laborious to TomoTherapy^®^ users since a separate DQA plan corresponding to each treatment plan needs to be created. Moreover, heavy devices are required on the treatment couch of the TomoTherapy^®^ system to perform the DQA measurements. It must be underlined that the DQAs performed with the above‐mentioned methods allow the dose comparison between the measurement and calculation only in the small region or the plane, where the measurement tools (i.e., the ionization chamber, film, detector array, etc.) are positioned, and require much work in cases where the volume to be treated extends beyond their physical dimensions. Therefore, with the current TomoTherapy^®^ DQA modalities, it is hard to identify the region accurately inside the patient body where non‐negligible dose difference between the measurement and calculation is present.

Recently, various new systems have been commercially released for DQA of patient treatment plans using modern radiotherapy modalities.^5^, ^7^ These systems use log‐files of the beam irradiation or measured beam fluence to reconstruct the dose distribution on the CT images of patients, thereby reconstructing the dose to the target and surrounding normal structures. These systems make the DQA analysis available not only in a point dose and two‐dimensional (2D) planar dose distribution but also in three‐dimensional (3D) volumetric dose distribution inside the patient body. Therefore, users can compare the dose distribution between the measurement and calculation in more detail compared with the traditional DQA methods.

The purpose of this work is to investigate the clinical suitability of a new commercial DQA system, Dosimetry Check™(DC, MathResolutions, LLC., Columbia, MD, USA), on TomoTherapy^®^‐based IMRT plans. The DQA tests have been performed for TomoTherapy^®^‐based clinical helical treatment plans covering various treatment sites. For the same treatment plans, the DQA process was also carried out using the traditional cheese phantom method. We compared the DQA results obtained with these two different methods in absolute dose difference and gamma pass rate of 2D planar dose distribution.

## 2 MATERIALS AND METHODS

### 2.A TomoTherapy^®^

The TomoTherapy^®^ unit is designed to provide intensity‐modulated radiotherapy delivery with flattening‐filter free 6 MV photon beam and binary 64 multileaf collimators (MLCs).^8^ In our clinic, two different TomoTherapy^®^ units were used for patient treatments: TomoTherapy^®^ HD and TomoTherapy^®^ Hi‐ART. TomoTherapy^®^ HD provides both helical and TomoDirect™ modes, while the TomoTherapy Hi‐ART provides only helical mode. These two TomoTherapy^®^ units clinically used three different field widths of 1.0, 2.5, and 5.0 cm, which were defined by jaws along the longitudinal direction.

TomoTherapy^®^‐based IMRT treatment plans are created by its own integrated treatment planning system (TPS).^2^ The TomoTherapy^®^ TPS provides inverse planning capability in the optimization process and determines the leaf positions for all the gantry angles and couch positions. The inverse planning process is carried out until all the dose constraints are satisfied or have been optimized. The final dose calculation is performed with a convolution/superposition algorithm.

### 2.B Dosimetry Check™

DC (version 5.2.4) is a software that carries out DQA by reconstructing 3D dose distribution on the CT images of a phantom or patient.^9^ For the DQA of TomoTherapy^®^ treatment plans, DC uses the measured beam fluence, i.e., the sinogram, as the radiation source for the dose reconstruction. The beam fluence is recorded by the TomoTherapy^®^ MVCT detector positioned in opposite side to the linear accelerator/target. The current DC software reconstructs the 3D dose distribution based on pencil‐beam (PB) algorithm or collapsed‐cone convolution (CCC) algorithm, while it only used PB algorithm in the previous version. In this work, we reconstructed the dose distribution in DC using CCC algorithm with a 5 mm grid size. DC carries out the DQA analysis using two different modes: pretreatment dosimetry mode and *in vivo* dosimetry mode. In the pretreatment dosimetry mode, no material is present inside the treatment unit bore except for the treatment couch. In the *in vivo* dosimetry mode, the phantom or patient is positioned inside the treatment bore. In this work, we tested the pretreatment dosimetry mode for the clinical application of DC. Since the *in vivo* dosimetry mode in DC was not commissioned in our institution, we excluded to test this mode in this work.

### 2.C Dose delivery verification

**Figure 1:**
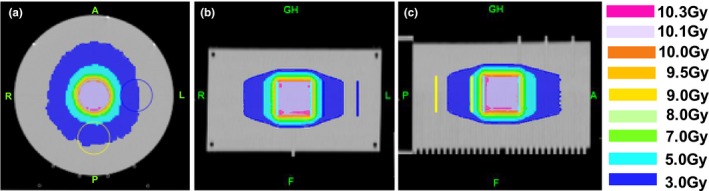
A treatment plan for TomoTherapy^®^‐based IMRT delivery verification in (a) axial, (b) coronal and (c) sagittal planes.

Before using it for clinical DQA, it is required to evaluate whether DC performs the dose reconstruction accurately as measured by the ionization chamber or not. By performing this process, we could reflect the output of the TomoTherapy^®^ treatment units to the DC‐based dose reconstruction. For this test, we used a TomoTherapy^®^‐based IMRT delivery verification plan, which was created on a cylindrical Solid Water™ phantom (i.e., the cheese phantom) by the TomoTherapy^®^ factory and used in acceptance test procedure (ATP) during the TomoTherapy^®^ treatment machine installation. Three different IMRT verification plans were created using the 1.0, 2.5, and 5.0 cm field widths. Each verification plan was generated to deliver uniform dose to the cylindrical target positioned at the center of the phantom as shown in Fig. 1. The prescription dose to the cylindrical target was 10 Gy in five fractions, where 95% of the target volume receiving at least 10 Gy. The dose distribution to the cheese phantom with each verification plan was reconstructed using DC in the pretreatment dosimetry mode. The actual dose delivered to the cylindrical phantom was also measured using an Exradin A1SL air‐filled thimble ionization chamber (Standard Imaging, Madison, WI), which was positioned 0.5 cm below the center of the cylindrical target volume. The Exradin A1SL was polarized by TomoElectrometer (Standard Imaging, Inc., Middleton, WI) with −300 V. The DC‐reconstructed dose at the same position, where the Exradin A1SL collecting volume was placed, was compared with the chamber‐measured dose for each verification plan.

### 2.D Patient‐specific DQA for TomoTherapy^®^‐based helical IMRT plans

The patient‐specific DQA test with DC was performed for 15 different TomoTherapy^®^‐based helical treatment plans, which were randomly selected from our institution's patient list. Seven treatment plans were delivered on the TomoTherapy^®^ HD unit and other eight treatment plans were delivered on the TomoTherapy^®^ Hi‐ART unit. The treatment sites of these clinical plans were prostate, brain, head and neck, lung, abdomen and spine. Most of the treatment plans were generated using the 2.5 cm field width except for one abdomen treatment plan, which was created using the 5.0 cm field width.

In the TomoTherapy^®^ treatment machine, a couch position was set to a height of 400 mm and each plan was delivered using QA‐Treatment mode. The treatment plan was archived and the recorded beam fluence from the TomoTherapy^®^ MVCT detector was saved as .xml format. Since the operating software version of our TomoTherapy^®^ units did not support the DC automation system, the beam fluence was manually converted to a Digital Imaging and Communications in Medicine (DICOM) format (.dcm) or .bin format using in‐house software and then transferred to the DC‐installed computer. Also, the entire treatment plan data in the DICOM format (i.e., CT images, RT Plan, RT Dose and RT Structure files) computed on TomoTherapy^®^ TPS were also transferred to the DC‐installed computer.

In DC software, a TomoTherapy^®^ couch was inserted and an external contour of the patient body was delineated on the CT image of the patient. Then, a reference volume with a 5 mm diameter was selected inside the GTV, CTV or PTV, where the uniform dose distribution was present as recommended by International Commission on Radiation Units and Measurements (ICRU) Report 62.^10^ The reference volume was defined to compare the DC‐calculated dose to the chamber‐measured value. The 5 mm diameter was chosen to mimic the dimension of the Exradin A1SL chamber collecting volume. Using the CCC algorithm in DC, the radiation dose distribution to the patient was reconstructed and compared with the TPS‐calculated dose distribution. Using Intel^®^ Core™ i7‐6900K CPU at 3.20 GHz, the dose reconstruction time in DC for each treatment plan was about 30 min with the CCC algorithm and 5 mm grid size. The DC‐reconstructed and TomoTherapy^®^ TPS‐calculated dose distributions were compared at a point dose as well as in 1D, 2D, and 3D methods. The 1D dose profile was compared in x‐, y‐, and z‐axes defined at the reference point. The 2D planar dose was compared in transverse, coronal and sagittal planes defined at the reference point using a gamma analysis method. The 3D volumetric comparison was performed using 3D gamma pass analysis. Both the 2D and 3D gamma pass analysis were performed using a 3%/3 mm dose difference/distance to agreement criteria^11^ as recommended by AAPM TG‐148 Report.^2^

The DQA process for each treatment plan was also performed using the traditional DQA method for TomoTherapy^®^ pretreatment verification. Each clinical treatment plan was transferred to the CT images of the cheese phantom in the TomoTherapy^®^ DQA workstation. The position of the phantom was moved to place the uniform dose distribution area (generally inside the target volume) near the center of the phantom, where the A1SL chamber was inserted during the DQA measurement. The dose measurement position with the ionization chamber was selected similar to the reference point where the absolute dose difference was calculated using DC. Then, the dose distribution to the cheese phantom was calculated. Inside the TomoTherapy^®^ unit bore, the cheese phantom was positioned with the Exradin A1SL ionization chamber and Gafchromic™ EBT3 radiochromic film (Ashland Advanced Materials, Bridgewater, NJ, USA) to measure the point dose and 2D planar dose distribution, respectively. The chamber was positioned 5 mm below the center of the cheese phantom and polarized with −300 V using the TomoElectrometer. The EBT3 film was positioned in coronal or sagittal direction at the middle of the cheese phantom. After the cheese phantom was irradiated with the DQA plan, the measured point dose and 2D planar dose distribution were compared with the calculated ones using the TomoTherapy^®^ DQA workstation.

## 3 RESULTS

### 3.A Dose delivery verification

In Table 1, the absolute dose differences of each IMRT verification plan between DC, TomoTherapy^®^ TPS and Exradin A1SL ionization chamber are reported for both TomoTherapy^®^ units considered in this study and for all field widths. For the TomoTherapy^®^ HD, the DC‐reconstructed dose was different from chamber‐measured dose by −0.5%, 0.0%, and 0.7% for 1.0, 2.5, and 5.0 cm field width plans, respectively. For the TomoTherapy^®^ Hi‐ART, the differences between the DC‐reconstructed and chamber‐measured doses were −1.1%, 1.6%, and 0.8% for 1.0, 2.5, and 5.0 cm field width plans, respectively. This dose difference is similar to the uncertainty of ionization chamber measurement, 0.9%, as analyzed by McEwen et al.^12^ However, since this dose difference may affect to the absolute dose difference and gamma pass rate of the DC‐based DQA analysis, we used this information for the absolute dose calibration of DC. The absolute dose calibration process was performed by providing the DC‐calculated and chamber‐measured doses in DC, which were used to modify the dose conversion constant and thereby matching the DC‐calculated dose to the chamber‐measured value.

**Table 1:** Comparison of Dosimetry Check™‐calculated, TomoTherapy^®^ TPS‐calculated and Exradin A1SL ionization chamber‐measured doses for the IMRT verification plan shown in Fig. 1

Field width (cm)	Machine	Absolute dose difference (%)	
		DC − Exradin A1SL	TomoTherapy TPS − Exradin A1SL
1.0	Tomo HD	−0.5	−0.8
	Tomo Hi‐Art	−1.1	0.4
2.5	Tomo HD	0.0	0.2
	Tomo Hi‐Art	1.6	1.5
5.0	Tomo HD	0.7	1.1
	Tomo Hi‐Art	0.8	1.5

### 3.B Patient‐specific DQA results for TomoTherapy^®^‐based helical IMRT plans

**Figure 2:**
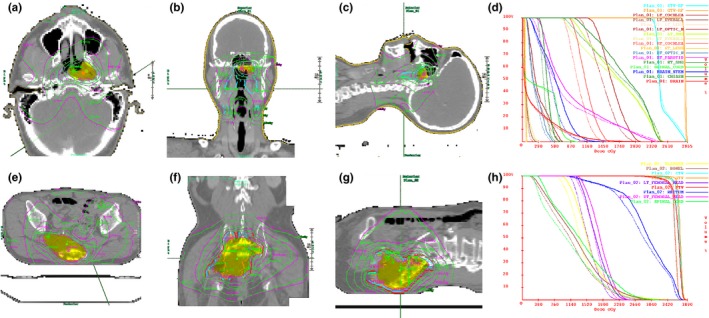
Examples of the DQA results using Dosimetry Check™ for (a)–(c) head and neck and (e)–(g) abdominal cancer patients. For each treatment plan, the 2D planar dose distributions are compared in (a), (e) axial, (b), (f) coronal and (c), (g) sagittal planes. The magenta and green isolines refer to DC and TomoTherapy^®^ TPS‐calculated dose distributions, respectively. Figures (d) and (h) present the dose–volume histogram of the target and normal structures calculated in Dosimetry Check™ (solid line) and TomoTherapy^®^ TPS (dashed line).

Figure 2 shows examples of DQA results obtained with DC for head and neck and abdominal cancer patients. Table 2 summarizes the DQA results obtained with the DC and cheese phantom methods for the 15 helical TomoTherapy^®^‐based clinical IMRT plans. In general, DC provided very similar DQA results to those measured with the cheese phantom method. For the TomoTherapy^®^ HD treatment plans, the absolute dose difference calculated at the reference point between the DC and TomoTherapy^®^ TPS ranged from 0.64% to 4.22%, while it ranged from −2.05% to 1.74% for the TomoTherapy^®^ Hi‐ART treatment plans. With the cheese phantom method, the absolute dose difference between the measurement with the Exradin A1SL chamber and the calculation with TomoTherapy^®^ DQA workstation ranged from −0.11% to 2.37% and from −1.89% to 1.40% for the TomoTherapy^®^ HD and Hi‐ART treatment plans, respectively. The *P*‐value calculated using Wilcoxon signed‐rank test was 0.256, which implies there is no statistically significant difference between the two DQA methods in determining the absolute dose difference.

**Table 2:** Summary of DQA results of the 15 clinical TomoTherapy^®^ treatment plans using the Dosimetry Check™ and cheese phantom method. The *P*‐value for the absolute dose difference determined by the two different DQA methods was calculated using Wilcoxon signed‐rank test

Patient no.	Treatment machine	Treatment site	Absolute dose difference (%)		Gamma pass rate (%)					
			Dosimetry Check™	Cheese phantom	Dosimetry Check™				Cheese phantom	
					Axial	Coronal	Sagittal	3D Body	Coronal	Sagittal
1	Tomo HD	Prostate	2.94	1.60	97.9	99.8	97.7	99.3	–	99.7
2		Head and neck	2.13	2.37	100	99.9	99.9	99.9	99.2	–
3			1.06	1.19	98.2	99.4	99.9	99.5	99.9	–
4			1.73	0.15	99.2	98.1	100	99.5	99.9	–
5		L‐spine	1.89	1.65	97.6	99.5	99.8	99.7	–	99.9
6		T‐spine	0.64	−0.11	99.5	99.7	99.1	99.7	99.5	–
7		Abdomen	4.22	0.38	46.5	82.2	87.8	84.2	–	99.6
8	Tomo Hi‐ART	Prostate	1.51	1.20	99.6	99.8	99.3	99.4	–	99.6
9		Head and neck	1.56	0.90	100	100	100	99.7	96.5	–
10			−0.28	1.40	97.8	100	99.9	99.8	99.7	–
11			1.04	0.80	98.1	99.4	98.2	99.0	99.8	–
12		Lung	−1.95	−1.89	99.4	99.3	99.5	98.9	97.6	–
13		Retroperitoneum	−1.93	0.60	95.6	98.4	98.9	99.2	–	96.8
14			−2.05	−1.35	96.0	98.2	98.1	98.2	–	96.0
15		Brain	1.74	−1.04	95.6	99.5	100	98.8	99.1	–
*P*‐value			0.256		N/A					

For 14 out of the 15 treatment plans where the treatment sites were prostate, head and neck, lung and L‐spine, the absolute dose difference was less than ±3% and the 2D and 3D gamma pass rates with the 3%/3 mm gamma criteria were greater than 95% with both DQA methods. However, for the abdomen treatment plan (Patient No. 7), it was shown that the absolute dose difference was greater than 3% and the gamma pass rate with the 3%/3 mm gamma criteria was less than 95% with DC. When using the cheese phantom, on the other hand, the DQA result for the same treatment plan passed our institution's tolerance, i.e., the absolute dose difference is less than 3% and the gamma pass rate with the 3%/3 mm gamma criteria is greater than 95% in 2D planar evaluation.

## 4 DISCUSSION

We have investigated the clinical usability of DC to verify the dose delivery in a pretreatment mode before the actual patient treatments. DC not only provides information of the absolute dose difference at the reference point and 2D planar gamma pass rate similar to the DQA results obtained with the cheese phantom method but also provides the 3D volumetric gamma pass rate and the comparison of dose–volume histogram (DVH) for defined target volumes and normal structures between the DC and TomoTherapy^®^ TPS. Furthermore, DC provides the 2D planar gamma pass rate in the axial, coronal and sagittal planes simultaneously, while the DQA method with the cheese phantom provides the 2D planar gamma pass rate only in the coronal or sagittal plane.

The clinical availability of DC has been investigated by various research groups for DQA of linear accelerator (LINAC) and TomoTherapy^®^‐based IMRT plans.^13^, ^14^, ^15^, ^16^, ^17^, ^18^ These studies reported that the DC‐calculated 3D volumetric gamma pass rates for the patient‐specific DQAs were greater than 90%. Mezzenga et al.^16^ reported that the gamma pass rate of the DC‐based DQA result decreased to about 90% when the grid size of dose reconstruction increased up to 8 mm. They recommended using a 5 mm grid size for the dose reconstruction in DC since it provided gamma pass rates greater than 95% with reasonable calculation time. In this work, we randomly selected TomoTherapy^®^‐based IMRT plans actually used for patient treatments covering various treatment sites. For 14 out of the 15 TomoTherapy^®^ treatment plans, we obtained gamma pass rates greater than 95% with the 3%/3 mm gamma criteria both in 2D planar and 3D volumetric evaluations using a 5.0 mm grid size. We analyzed the gamma pass rates not only in 3D volume but also in the 2D plane in DC. This makes the direct comparison of the DQA results obtained with the DC and cheese phantom in the same dimension. In this work, we did not find any dependency of the DQA result with the DC on the TomoTherapy^®^ treatment machine as we tested two different machines simultaneously.

Most of the TomoTherapy^®^ treatment plans used in this work showed very similar DQA results between the DC and cheese phantom method. However, only for one treatment plan (Patient No. 7, which was computed using the 5.0 cm field width), the absolute dose difference was greater than 3% and the gamma pass rate was less than 95% with the 3%/3 mm gamma criteria with DC. Even though not listed in this work, we also observed the DC results did not pass our institution's TomoTherapy^®^ DQA tolerance for other 5.0 cm field width‐used treatment plans such as breast, abdomen, esophagus, pelvic bone, etc. This can be considered as the limitation in using DC for the DQA of the TomoTherapy^®^‐based helical treatment plans. When the target volume was significantly long in the longitudinal direction, we used the 5.0 cm field width for generating helical TomoTherapy^®^ treatment plans to reduce the treatment time. Sometimes, the shape of target volume was also irregular, thereby increasing MLC modulation factor to achieve the desired dose distribution to the target volume. In our institution, as an alternative method, we use the traditional cheese phantom method for the DQA of 5.0 cm field width‐used treatment plans. Further study is required to investigate the reason of unsatisfying DC‐based DQA results for the TomoTherapy^®^ treatment plans using the 5.0 cm field width.

In DC software used in this work (version 5.2.4), it was not possible to analyze the gamma pass rate to a specific region of interest especially in 2D plane. Therefore, in this work, we could not but analyze the gamma pass rate in the entire area instead of restricting to the target volume. In this case, the gamma pass rate could be overestimated since the gamma evaluation includes low‐dose regions outside the target volume. Therefore, when reviewing DC‐based DQA results, we would like to recommend examine carefully the profiles near the target volume, not the gamma pass rate only.

Sometimes the measured beam fluence included the noise signal at starting and ending of the beam irradiation. Since the measured beam fluence also included the information of the gantry angle position for the corresponding beam intensity, it was found the DC‐reconstructed dose distribution was rotated compared to the TomoTherapy^®^ TPS‐calculated one for some treatment plans. For this case, using another in‐house software provided by the DC manufacturer, the measured beam fluence was trimmed by artificially deleting the noise signal. When applying the trimmed beam fluence as the radiation source, we observed that the rotated dose distribution was fixed to the correct one (Patients No. 6, 14, and 15) and the gamma pass rates were greater both in the 2D and 3D evaluations. Therefore, if the DC‐reconstructed dose distribution was rotated and significantly different from the TPS‐calculated one, it is strongly recommended to investigate whether the trimmed beam fluence file is generated or not using the in‐house software and then use the trimmed one for the dose reconstruction in DC.

Since DC can reconstruct the dose distribution to the CT image of the patient, it is also available to compare the DVH for the targets and surrounding normal structures between the TomoTherapy^®^ TPS and DC. It is possible to have discrepancies between the TomoTherapy^®^ TPS and DC‐calculated DVHs for target or normal structures even though the 3D volumetric gamma pass rates for the corresponding structures satisfy the gamma pass criteria. Especially when the tissue inhomogeneity is present in the target or surrounding normal structures, the dose distribution difference can be present due to the inherent difference of the dose calculation algorithm between the CCC algorithm in DC and convolution/superposition algorithm in TomoTherapy^®^ TPS.^17^ Users may need to determine the acceptable limit of the discrepancy about the DVH between the TomoTherapy^®^ TPS and DC.

Based on our institution's experience, DC shortened the processing time of patient‐specific DQA for TomoTherapy^®^‐based helical treatment plans. When using DC, no DQA plan or additional device is necessary likely to the cheese phantom method, thereby reducing the DQA preparation time and requiring less human resources. In a treatment room, only couch positioning and beam irradiation time slot is required, which is around 10–15 min per treatment plan including the measured beam fluence data transfer to a DC‐installed computer. Including the dose reconstruction processing time, we could complete overall process of the DC‐based DQA analysis within 1 h per treatment plan. Even though automation program of DC was not used in this work because of the technical issue, we expect that the automation program will accelerate the overall DQA process since the measured beam fluence transfer as well as dose reconstruction process can be automatically performed.

In this work, since DC installed in our institution was primarily used for the DQA before the patient treatment, it was only commissioned on the pretreatment dosimetry mode. As mentioned by Mezzenga et al.^16^ and Gimeno et al.,^18^ DC also can be used on the *in vivo* dosimetry mode, where the beam fluence is measured by the TomoTherapy^®^ MVCT detector with the presence of the phantom or the patient inside the treatment bore. Even though the separate commissioning is required for the *in vivo* dosimetry mode, DC might be available to monitor variation of the dose delivery to the target and normal structures for each treatment fraction, thereby qualifying the dose delivery to the patient more accurately. The validation of DC in the *in vivo* dosimetry mode would be a part of our future study.

## 5 CONCLUSION

In this work, we have clinically implemented DC for the DQA of TomoTherapy^®^‐based helical IMRT plans. DC generally carried out accurate DQA results for treatment plans covering various treatment sites as performed by the traditional cheese phantom method. Furthermore, DC could provide more information in verifying the dose delivery to the patient, while simplifying the DQA process. Based on the comparison of the DQA results between DC and traditional cheese phantom method, we concluded that DC is clinically available for patient‐specific DQA of TomoTherapy^®^‐based helical IMRT plans. Currently, DC is used as the primary resource of the DQA for TomoTherapy^®^‐based helical IMRT plans in our institution.

## CONFLICT OF INTEREST

Conflict of interest relevant to this article was not reported.

## References

[1] Fenwick JD , Tome WA , Jaradat HA , et al. Quality assurance of a helical tomotherapy machine. Phys Med Biol. 2004;49:2933–2953. 15285257 10.1088/0031-9155/49/13/012

[2] Langen KM , Papanikolaou N , Balog J , et al. QA for helical tomotherapy: report of the AAPM Task Group 148. Med Phys. 2010;37:4817–4853. 20964201 10.1118/1.3462971

[3] Feygelman V , Opp D , Javedan K , Saini AJ , Zhang G . Evaluation of a 3D diode array dosimeter for helical tomotherapy delivery QA. Med Dosim. 2010;35:324–329. 20097061 10.1016/j.meddos.2009.10.007

[4] Myers P , Stathakis S , Gutierrez AN , Esquivel C , Mavroidis P , Papanikolaou N . Evaluation of PTW Seven29 for tomotherapy patient‐specific quality assurance and comparison with ScandiDos Delta(4). J Med Phys. 2012;37:72–80. 22557796 10.4103/0971-6203.94741 PMC3339146

[5] Clemente‐Gutierrez F , Perez‐Vara C . Dosimetric validation and clinical implementation of two 3D dose verification systems for quality assurance in volumetric‐modulated arc therapy techniques. J Appl Clin Med Phys. 2015;16:5190. 26103189 10.1120/jacmp.v16i2.5190 PMC5690088

[6] Xu S , Xie C , Ju Z , et al. Dose verification of helical tomotherapy intensity modulated radiation therapy planning using 2D‐array ion chambers. Biomed Imaging Interv J. 2010;6:e24. 21611040 10.2349/biij.6.2.e24 PMC3097769

[7] Nelson CL , Mason BE , Robinson RC , Kisling KD , Kirsner SM . Commissioning results of an automated treatment planning verification system. J Appl Clin Med Phys. 2014;15:4838. 25207567 10.1120/jacmp.v15i5.4838 PMC5711088

[8] Mackie TR , Holmes T , Swerdloff S , et al. Tomotherapy: a new concept for the delivery of dynamic conformal radiotherapy. Med Phys. 1993;20:1709–1719. 8309444 10.1118/1.596958

[9] Renner WD . 3D dose reconstruction to insure correct external beam treatment of patients. Med Dosim. 2007;32:157–165. 17707194 10.1016/j.meddos.2007.02.005

[10] International Organization of Radiation Units and Measurements . ICRU Report 62: Prescribing, recording and reporting photon beam therapy (supplement to ICRU Report 50). Bethesda, MD: International Commission on Radiation Units and Measurements; 1999.

[11] Low DA , Harms WB , Mutic S , Purdy JA . A technique for the quantitative evaluation of dose distributions. Med Phys. 1998;25:656–661. 9608475 10.1118/1.598248

[12] McEwen M , DeWerd L , Ibbott G , et al. Addendum to the AAPM's TG‐51 protocol for clinical reference dosimetry of high‐energy photon beams. Med Phys. 2014;41:041501. 24694120 10.1118/1.4866223 PMC5148035

[13] Nelms BE , Zhen H , Tome WA . Per‐beam, planar IMRT QA passing rates do not predict clinically relevant patient dose errors. Med Phys. 2011;38:1037–1044. 21452741 10.1118/1.3544657 PMC3188652

[14] Wu C , Hosier KE , Beck KE , et al. On using 3D gamma‐analysis for IMRT and VMAT pretreatment plan QA. Med Phys. 2012;39:3051–3059. 22755690 10.1118/1.4711755

[15] Liang Y , Kim GY , Pawlicki T , Mundt AJ , Mell LK . Feasibility study on dosimetry verification of volumetric‐modulated arc therapy‐based total marrow irradiation. J Appl Clin Med Phys. 2013;14:3852. 23470926 10.1120/jacmp.v14i2.3852 PMC5714362

[16] Mezzenga E , Cagni E , Botti A , et al. Pre‐treatment and in‐vivo dosimetry of Helical Tomotherapy treatment plans using the Dosimetry Check system. J Instrum. 2014;9:C04039.

[17] Narayanasamy G , Zalman T , Ha CS , Papanikolaou N , Stathakis S . Evaluation of Dosimetry Check software for IMRT patient‐specific quality assurance. J Appl Clin Med Phys. 2015;16:5427. 26103501 10.1120/jacmp.v16i3.5427 PMC5690116

[18] Gimeno J , Pujades MC , Garcia T , et al. Commissioning and initial experience with a commercial software for in vivo volumetric dosimetry. Phys Med. 2014;30:954–959. 24998334 10.1016/j.ejmp.2014.06.004

